# Supplementary material for: The genetic architecture of the load linked to dominant and recessive self-incompatibility alleles in Arabidopsis halleri and Arabidopsis lyrata
Source: eLife. 2024 Sep 2;13:RP94972. doi: 10.7554/eLife.94972 (PMC11368402; doi:10.7554/eLife.94972)
Supplement: Supplementary file 1. — (a) Crosses performed to obtain homozygotes for three S-alleles. (b) Trait variation in S-locus homozygous individuals. (c) Trait variation in homozygous at the S-locus for the S-alleles Ah01 and Ah04 between families. (d) Effect of dominance on variation of phenotypic traits in S-locus homozygous individuals. (e) Number of phased haplotypes linked to S-alleles in each sample. (f) Effect of dominance on the accumulation of genetic load in S-flanking regions. (g) S-locus genotypes of individuals sequenced using the capture protocol. (h) S-locus genotypes of the offspring selected for haplotype phasing and the crosses for the study of phenotypic traits. (i) Effect of phytopathogen, phytophagous attacks and oxidative stress on the phenotypic traits. (j) Difference on the phenotypic traits variation between the two families for each allele tested. [file elife-94972-supp1.docx]

***Supplementary file 1a: Crosses performed to obtain homozygotes for three S-alleles.*** *For each S-allele, we obtained homozygous and heterozygous plants within two separate families (listed as separate rows).*

| **Genotype pollen donor ^a^** | **Genotype stigma recipient** | **Homozygote studied** | **Dominance level** | **Number of fruits obtained** | **Number of seeds** |
| --- | --- | --- | --- | --- | --- |
| **Ah20**/Ah01 | Ah12/Ah01 | Ah01 | 1 | 5 | 48 |
| **Ah20**/Ah01 | Ah12/Ah01 | Ah01 | 1 | 2 | 23 |
| **Ah02**/Ah03 | Ah03/Ah01 | Ah03 | 2 | 6 | 69 |
| **Ah24**/Ah03 | Ah03/Ah01 | Ah03 | 2 | 3 | 27 |
| **Ah20**/Ah04 | Ah04/Ah04 | Ah04 | 3 | 21 | 167 |
| **Ah20**/Ah04 | Ah04/Ah04 | Ah04 | 3 | 8 | 65 |

*^a^* *The S-alleles in bold represent the dominant S-allele expressed on pollen of each donor genotype. Each line corresponds to one pair of individuals used as parents.*

***Supplementary file 1b: Trait variation in S-locus homozygous individuals.***

| **Trait (unit)** | **Mean heterozygotes**  **(SD)** | **Mean homozygotes**  **(SD)** | **P value ^a^** |
| --- | --- | --- | --- |
| Time first leaf (day) | 15.7 (1.04) | 14.79 (1.19) | 0.14 |
| Number of leaves | 13.22 (0.65) | 13.46 (0.96) | 0.35 |
| Rosette area (cm²) | 34.99 (5.11) | 35.5 (7.65) | 0.46 |
| Mean leaf length (cm) | 1.86 (0.15) | 1.86 (0.21) | 0.49 |
| St dev leaf length (cm) | 0.28 (0.02) | 0.29 (0.04) | 0.31 |
| Mean leaf width (cm) | 1.49 (0.11) | 1.46 (0.14) | 0.36 |
| St dev leaf width (cm) | 0.27 (0.02) | 0.25 (0.03) | 0.24 |
| Mean leaf area (cm²) | 3.16 (0.43) | 3.11 (0.6) | 0.45 |
| Time first flower (day) | 36.28 (1.14) | 34.98 (1.25) | 0.07 |
| Number of flowering stems | 12.9 (0.91) | 13.3 (1.32) | 0.31 |
| **Maximum flowering stem length (cm)** | **56.19 (2.26)** | **64.55 (3.17)** | **<1e^-04^** |
| Number of flowers by flowering stem | 58.87 (5.96) | 66.56 (10.71) | 0.09 |
| Flowering duration (day) | 44.15 (1.7) | 43.81 (1.88) | 0.39 |

*^a^ The P value represents the proportions of the distribution with the expected difference of mean between heterozygotes and homozygotes at the S-locus after 10,000 random resamples equal to or less than the value observed. Significant values are represented in bold.*

***Supplementary file 1c: Trait variation in homozygous at the S-locus for the S-alleles Ah01 and Ah04 between families.***

| **Allele** | **Trait (unit)** | **Mean first family (SD)** | **Mean second family (SD)** | **p value** |
| --- | --- | --- | --- | --- |
| Ah01^b^ | **Time first leaf (day)** | **17.11 ( 2.26 )** | **32 ( NA )** | **<1.00^e-04^** |
|  | Number of leaves | 14 ( 2.83 ) | 9 ( NA ) | 0.10 |
|  | Rosette area (cm²) | 27.65 ( 18.22 ) | 14.21 ( NA ) | 0.50 |
|  | Mean leaf length (cm) | 1.64 ( 0.68 ) | 1.52 ( NA ) | 0.49 |
|  | St dev leaf length (cm) | 0.28 ( 0.11 ) | 0.17 ( NA ) | 0.30 |
|  | Mean leaf width (cm) | 1.38 ( 0.43 ) | 1.38 ( NA ) | 0.49 |
|  | St dev leaf width (cm) | 0.24 ( 0.07 ) | 0.16 ( NA ) | 0.30 |
|  | Mean leaf area (cm²) | 2.52 ( 1.59 ) | 2.1 ( NA ) | 0.50 |
|  | Time first flower (day) | 35.86 ( 3.13 ) | 36 ( NA ) | 0.25 |
|  | Number of flowering stems | 12.29 ( 3.9 ) | 7 ( NA ) | 0.25 |
|  | **Maximum flowering stem length (cm)** | **43.43 ( 6.91 )** | **62.4 ( NA )** | **<1.00^e-04^** |
|  | Number of flowers by flowering stem | 31.73 ( 24.16 ) | 52.25 ( NA ) | 0.12 |
|  | **Flowering duration (day)** | **44.71 ( 6.73 )** | **52 ( NA )** | **<1.00^e-04^** |
| Ah04 | Time first leaf (day) | 13.15 ( 2.68 ) | 14.88 ( 4.36 ) | 0.11 |
|  | Number of leaves | 13.43 ( 2.99 ) | 14.04 ( 3.9 ) | 0.24 |
|  | Rosette area (cm²) | 36.63 ( 27.74 ) | 45.86 ( 33.48 ) | 0.08 |
|  | Mean leaf length (cm) | 1.93 ( 0.81 ) | 2.13 ( 0.89 ) | 0.14 |
|  | St dev leaf length (cm) | 0.29 ( 0.15 ) | 0.3 ( 0.12 ) | 0.33 |
|  | Mean leaf width (cm) | 1.54 ( 0.57 ) | 1.44 ( 0.51 ) | 0.20 |
|  | **St dev leaf width (cm)** | **0.28 ( 0.13 )** | **0.22 ( 0.09 )** | **0.02** |
|  | Mean leaf area (cm²) | 3.4 ( 2.45 ) | 3.46 ( 2.27 ) | 0.45 |
|  | Time first flower (day) | 35.57 ( 4.73 ) | 33.2 ( 3.66 ) | 0.06 |
|  | **Number of flowering stems** | **13.21 ( 4.25 )** | **16.4 ( 5.25 )** | **0.02** |
|  | Maximum flowering stem length (cm) | 64.98 ( 9.65 ) | 62.41 ( 9.34 ) | 0.13 |
|  | **Number of flowers by flowering stem** | **72.38 ( 33.07 )** | **45.25 ( 15.45 )** | **1.00^e-04^** |
|  | Flowering duration (day) | 43.68 ( 6.96 ) | 41.56 ( 7.38 ) | 0.23 |

*^a^ The P value represents the proportions of the distribution with the expected difference of mean between each family after 10,000 random resamples equal to or less than the observed value. Significant values are represented in bold. The Ah03 S-allele is excluded because only two homozygotes survived. ^b^ the standard deviation for the second family was not estimated because only one homozygote survived.*

***Supplementary file 1d: Effect of dominance on variation of phenotypic traits in S-locus homozygous individuals.***

| **Trait (unit)** | **Random effect implemented in GLM model** | **Distribution of trait tested** | **Linear effect** | **p value** |
| --- | --- | --- | --- | --- |
| **Time first leaf (days)** | **Family** | **Poisson** | **-0.15** | **5.5^e-04^** |
| Leaves (counts) | Oxydative stress | Poisson | 0.006 | 0.91 |
| Rosette area (cm²) | Oxydative stress + family | Gaussian | 8.25 | 0.19 |
| Mean leave length (cm) | Oxydative stress + family | Gaussian | 0.17 | 0.28 |
| St dev leave length (cm) | None | Gaussian | 0.02 | 0.54 |
| Mean leave width (cm) | Oxydative stress + phytophagous attack | Gaussian | 0.05 | 0.83 |
| St dev leave width (cm²) | Oxydative stress + phytophagous attack | Gaussian | 0.01 | 0.91 |
| Mean leave area (cm²) | Oxydative stress | Gaussian | 0.39 | 0.36 |
| Time first flower (days) | Phytopathogen + phytophagous attack + family | Poisson | -0.0002 | 1 |
| Flowering stems (counts) | Family | Poisson | 0.15 | 0.08 |
| Maximum flowering stem length (cm) | Phytopathogen + family | Gaussian | 7.6 | 0.06 |
| Flowers by stem (counts) | Family | Poisson | 0.15 | 0.44 |
| Flowering duration (days) | Phytopathogen + family | Poisson | -0.05 | 0.1 |

*Dominance at the S-locus was implemented as a fixed effect. The dominance was considered a continuous variable.*

***Supplementary file 1e: Number of phased haplotypes linked to S-alleles in each sample.***

| **Species** | **Population** | **Number of phased haplotypes** | **Number of *S*-alleles** | **Number of *S-*alleles with more than one copy** | **Mean number of gene copies per *S*-allele ^a^** |
| --- | --- | --- | --- | --- | --- |
| *A. halleri* | Nivelle | 34 | 12 | 7 | 4.1 |
|  | Mortagne | 38 | 11 | 9 | 4 |
| *A. lyrata* | TSS | 16 | 5 | 3 | 4.7 |
|  | IND | 16 | 6 | 4 | 3.5 |
|  | PIN | 22 | 7 | 3 | 6 |

^a^ *based on S-alleles with more than one copy.*

***Supplementary file 1f: Effect of dominance on the accumulation of genetic load in S-flanking regions.***

| **Type of mutations** | **Species** | **Number of variable sites** | **Genetic load estimated** | **Linear effect** | **p value** |
| --- | --- | --- | --- | --- | --- |
| 0fold | *A. halleri* | 369 | **Number of fixed mutations by *S*-allele** | **0.08** | **2.57^e-04^** |
|  |  |  | **Number of segregating mutations by *S*-allele** | **-0.49** | **2.21^e-03^** |
|  |  |  | Mean total number of mutations by *S*-allele (S_0f_) | 0.01 | 0.54 |
|  | *A. lyrata* | 243 | **Number of fixed mutations by *S*-allele** | **0.08** | **4.50^e-03^** |
|  |  |  | Number of segregating mutations by *S*-allele | -0.44 | 0.09 |
|  |  |  | Mean total number of mutations by *S*-allele (S_0f_) | -0.07 | 0.07 |
| 4fold | *A. halleri* | 292 | **Number of fixed mutations by *S*-allele** | **0.23** | **8.95^e-04^** |
|  |  |  | **Number of segregating mutations by *S*-allele** | **-0.48** | **2.02^e-10^** |
|  |  |  | Mean total number of mutations by *S*-allele (S_4f_) | 1.53^e-03^ | 0.95 |
|  | *A. lyrata* | 189 | **Number of fixed mutations by *S*-allele** | **0.23** | **8.95^e-04^** |
|  |  |  | **Number of segregating mutations by *S*-allele** | **-0.46** | **0.04** |
|  |  |  | Mean total number of mutations by *S*-allele (S_4f_) | -0.04 | 0.32 |
| S_0f_/S_4f_ | *A. halleri* |  | Ratio | 0.02 | 0.42 |
|  | *A. lyrata* |  | Ratio | -0.11 | 0.11 |
| Non-synonymous | *A. halleri* | 376 | **Number of fixed mutations by *S*-allele** | **0.10** | **6.36^e-05^** |
|  |  |  | **Number of segregating mutations by *S*-allele** | **-0.09** | **2.98^e-03^** |
|  |  |  | Mean total number of mutations by S-allele (S_NS_) | 0.03 | 0.07 |
|  | *A. lyrata* | 250 | **Number of fixed mutations by *S*-allele** | **0.24** | **1.74^e-08^** |
|  |  |  | Number of segregating mutations by S-allele | -0.01 | 0.89 |
|  |  |  | **Mean total number of mutations by *S*-allele (S_NS_)** | **-0.11** | **2.06^e-16^** |
| Synonymous | *A. halleri* | 546 | **Number of fixed mutations by *S*-allele** | **0.10** | **4.36^e-07^** |
|  |  |  | **Number of segregating mutations by *S*-allele** | **-0.12** | **3.74^e-07^** |
|  |  |  | **Mean total number of mutations by *S*-allele (S_S_)** | **0.03** | **0.02** |
|  | *A. lyrata* | 369 | **Number of fixed mutations by *S*-allele** | **0.27** | **2.3^e-11^** |
|  |  |  | **Number of segregating mutations by *S*-allele** | **-0.17** | **3.42^e-03^** |
|  |  |  | **Mean total number of mutations by *S*-allele (S_S_)** | **-0.11** | **3.06^e-25^** |
| S_NS_/S_S_ | *A. halleri* |  | Ratio | -5.82^e-03^ | 0.81 |
|  | *A. lyrata* |  | Ratio | 0.02 | 0.60 |
| Deleterious SIFT4G | *A. halleri* | 58 | Number of fixed mutations by *S*-allele | 0.28 | 0.06 |
|  |  |  | Number of segregating mutations by S-allele | 0.01 | 0.47 |
|  |  |  | Mean total number of mutations by *S*-allele (S_D_) | 0.02 | 0.69 |
|  | *A. lyrata* | 33 | **Number of fixed mutations by *S*-allele** | **0.66** | **3.36^e-06^** |
|  |  |  | **Number of segregating mutations by S-allele** | **-0.05** | **0.01** |
|  |  |  | Mean total number of mutations by *S*-allele (S_D_) | -0.10 | 5.48^e-02^ |
| Lowly deleterious SNPeff | *A. halleri* | 548 | **Number of fixed mutations by *S*-allele** | **0.09** | **1.34^e-06^** |
|  |  |  | **Number of segregating mutations by *S*-allele** | **-0.12** | **1.46^e-06^** |
|  |  |  | **Mean total number of mutations by *S*-allele (S_LD_)** | **0.03** | **0.02** |
|  | *A. lyrata* | 373 | **Number of fixed mutations by *S*-allele** | **0.17** | **1.34^e-13^** |
|  |  |  | **Number of segregating mutations by *S*-allele** | **-0.17** | **0.47^E-03^** |
|  |  |  | **Mean total number of mutations by *S*-allele (S_LD_)** | **-0.11** | **3.51^e-25^** |
| Moderately deleterious SNPeff | *A. halleri* | 376 | **Number of fixed mutations by *S*-allele** | **0.11** | **1.31^e-05^** |
|  |  |  | **Number of segregating mutations by *S*-allele** | **-0.10** | **6.39^e-06^** |
|  |  |  | Mean total number of mutations by *S*-allele (S_MD_) | 0.03 | 0.07 |
|  | *A. lyrata* | 247 | **Number of fixed mutations by *S*-allele** | **0.13** | **4.45^e-06^** |
|  |  |  | Number of segregating mutations by *S*-allele | -0.02 | 0.74 |
|  |  |  | **Mean total number of mutations by *S*-allele (S_MD_)** | **-0.11** | **2.21^e-16^** |

*Dominance at the S-locus was implemented as a fixed effect. Populations were implemented as random effects. The distribution of the genetic load estimated followed a Poisson distribution, except the ratios that followed normal distributions.*

***Supplementary file 1g: S-locus genotypes of individuals sequenced using the capture protocol.***

| **Individual** | **Allele 1^a^** | **Allele 2^a^** | **Population** | **Species** | **SRA** |
| --- | --- | --- | --- | --- | --- |
| Mor_19_13 | Ah04 (III) | Ah25 (III) | Mortagne | *A. halleri* | SAMN20088356 |
| Mor_19_14 | Ah04 (III) | Ah01 (I) | Mortagne | *A. halleri* | SAMN20844087 |
| Mor_19_19 | Ah12 (IV) | Ah36 (IV) | Mortagne | *A. halleri* | SAMN20088358 |
| Mor_19_2 | Ah03 (II) | Ah24 (IV) | Mortagne | *A. halleri* | SAMN20088359 |
| Mor_19_23 | Ah20 (IV) | Ah25 (III) | Mortagne | *A. halleri* | SAMN20844088 |
| Mor_19_24 | Ah12 (IV) | Ah24 (IV) | Mortagne | *A. halleri* | SAMN20088362 |
| Mor_19_3 | Ah03 (II) | Ah01 (I) | Mortagne | *A. halleri* | SAMN20844098 |
| Mor_19_37 | Ah59 (IV) | Ah20 (IV) | Mortagne | *A. halleri* | SAMN20088357 |
| Mor_19_38 | Ah36 (IV) | Ah01 (I) | Mortagne | *A. halleri* | SAMN20088371 |
| Mor_19_4 | Ah25 (III) | Ah12 (IV) | Mortagne | *A. halleri* | SAMN20088360 |
| Mor_19_41 | Ah25 (III) | Ah25 (III) | Mortagne | *A. halleri* | SAMN20088372 |
| Mor_19_42 | Ah12 (IV) | Ah04 (III) | Mortagne | *A. halleri* | SAMN20088373 |
| Mor_19_45 | Ah36 (IV) | Ah02 (III) | Mortagne | *A. halleri* | SAMN20088374 |
| Mor_19_46 | Ah03 (II) | Ah12 (IV) | Mortagne | *A. halleri* | SAMN20088375 |
| Mor_19_51 | Ah20 (IV) | Ah05 (IV) | Mortagne | *A. halleri* | SAMN20088361 |
| Mor_19_53 | Ah36 (IV) | Ah12 (IV) | Mortagne | *A. halleri* | SAMN20088365 |
| Mor_19_54 | Ah24 (IV) | Ah12 (IV) | Mortagne | *A. halleri* | SAMN20088366 |
| Mor_19_55 | Ah20 (IV) | Ah05 (IV) | Mortagne | *A. halleri* | SAMN20088377 |
| Mor_19_56 | Ah12 (IV) | Ah03 (II) | Mortagne | *A. halleri* | SAMN20088378 |
| Niv_19_18 | Ah15 (IV) | Ah03 (II) | Nivelle | *A. halleri* | SAMN20088349 |
| Niv_19_19 | Ah20 (IV) | Ah24 (IV) | Nivelle | *A. halleri* | SAMN20088350 |
| Niv_19_22 | Ah24 (IV) | Ah01 (I) | Nivelle | *A. halleri* | SAMN20088351 |
| Niv_19_23 | Ah20 (IV) | Ah12 (IV) | Nivelle | *A. halleri* | SAMN20088352 |
| Niv_19_3 | Ah04 (III) | Ah59 (IV) | Nivelle | *A. halleri* | SAMN20088336 |
| Niv_19_31 | Ah04 (III) | Ah24 (IV) | Nivelle | *A. halleri* | SAMN20088337 |
| Niv_19_4 | Ah01 (I) | Ah24 (IV) | Nivelle | *A. halleri* | SAMN20088382 |
| Niv_19_42 | Ah22 (IV) | Ah12 (IV) | Nivelle | *A. halleri* | SAMN20088341 |
| Niv_19_45 | Ah12 (IV) | Ah04 (III) | Nivelle | *A. halleri* | SAMN20088344 |
| Niv_19_5 | Ah25 (III) | Ah67 (IV) | Nivelle | *A. halleri* | SAMN20088340 |
| Niv_19_52 | Ah20 (IV) | Ah22 (IV) | Nivelle | *A. halleri* | SAMN20088331 |
| Niv_19_53 | Ah05 (IV) | Ah20 (IV) | Nivelle | *A. halleri* | SAMN20088339 |
| Niv_19_54 | Ah01 (I) | Ah12 (IV) | Nivelle | *A. halleri* | SAMN20844097 |
| Niv_19_58 | Ah02 (III) | Ah59 (IV) | Nivelle | *A. halleri* | SAMN20088343 |
| Niv_19_59 | Ah01 (I) | Ah67 (IV) | Nivelle | *A. halleri* | SAMN20844096 |
| Niv_19_60 | Ah04 (III) | Ah20 (IV) | Nivelle | *A. halleri* | SAMN20088332 |
| Niv_19_7 | Ah04 (III) | Ah67 (IV) | Nivelle | *A. halleri* | SAMN20088345 |
| Niv_19_8 | Ah04 (III) | Ah12 (IV) | Nivelle | *A. halleri* | SAMN20844090 |
| Niv_19_9 | Ah04 (III) | Ah67 (IV) | Nivelle | *A. halleri* | SAMN20088338 |
| Pin_15_1 | Ah03 (II) | Ah01 (I) | PIN | *A. lyrata* | SAMN20088324 |
| Pin_16_1 | Ah01 (I) | Ah46 (IV) | PIN | *A. lyrata* | SAMN20088318 |
| Pin_4_24 | Ah03 (II) | Ah29 (III) | PIN | *A. lyrata* | SAMN20088314 |
| Pin_16_3 | Ah18 (IV) | Ah29 (III) | PIN | *A. lyrata* | SAMN20088323 |
| Pin_4_54 | Ah03 (II) | Ah03 (II) | PIN | *A. lyrata* | SAMN20088319 |
| Pin_5_1 | Ah01 (I) | Ah01 (I) | PIN | *A. lyrata* | SAMN20844102 |
| Pin_5_12 | Ah29 (III) | Ah01 (I) | PIN | *A. lyrata* | SAMN20088325 |
| Pin_5_2 | Ah03 (II) | Ah01 (I) | PIN | *A. lyrata* | SAMN20088311 |
| Pin_8_15 | Ah01 (I) | Ah01 (I) | PIN | *A. lyrata* | SAMN20088328 |
| Pin_8_2 | Ah29 (III) | Ah63 (III) | PIN | *A. lyrata* | SAMN20088326 |
| Pin_9_1 | Ah42 (IV) | Ah29 (III) | PIN | *A. lyrata* | SAMN20088312 |
| Tss_14_3 | Ah01 (I) | Ah31 (IV) | TSS | *A. lyrata* | SAMN20088315 |
| Tss_21_10 | Ah01 (I) | Ah03 (II) | TSS | *A. lyrata* | SAMN20088329 |
| Tss_22_24 | Ah01 (I) | Ah31 (IV) | TSS | *A. lyrata* | SAMN20088306 |
| Tss_22_7 | Ah01 (I) | Ah31 (IV) | TSS | *A. lyrata* | SAMN20088317 |
| Tss_3_10 | Ah31 (IV) | Ah01 (I) | TSS | *A. lyrata* | SAMN20088330 |
| Tss_23_2 | Ah03 (II) | Ah63 (III) | TSS | *A. lyrata* | SAMN20088313 |
| Tss_3_23 | Ah18 (IV) | Ah01 (I) | TSS | *A. lyrata* | SAMN20088307 |
| Tss_5_1 | Ah01 (I) | Ah31 (IV) | TSS | *A. lyrata* | SAMN20088316 |
| Ind_1_1 | Ah01 (I) | Ah01 (I) | IND | *A. lyrata* | SAMN20088320 |
| Ind_10_3 | Ah31 (IV) | Ah24 (IV) | IND | *A. lyrata* | SAMN20088327 |
| Ind_15_1 | Ah03 (II) | Ah24 (IV) | IND | *A. lyrata* | SAMN20088321 |
| Ind_15_2 | Ah63 (III) | Ah03 (II) | IND | *A. lyrata* | SAMN20088309 |
| Ind_9_3 | Ah01 (I) | Ah46 (IV) | IND | *A. lyrata* | SAMN20088322 |
| Ind_18_1 | Ah01 (I) | Ah01 (I) | IND | *A. lyrata* | SAMN20844103 |
| Ind_6_1 | Ah01 (I) | Ah63 (III) | IND | *A. lyrata* | SAMN20088308 |
| Ind_8_1 | Ah03 (II) | Ah63 (III) | IND | *A. lyrata* | SAMN20088305 |

*^a^* *The individuals were mainly sequenced by capture approach. The genotypes of homozygotes were confirmed after a whole genome sequencing. The class of dominance is specified in parenthesis.*

***Supplementary file 1h: S-locus genotypes of the offspring selected for haplotype phasing and the crosses for the study of phenotypic traits.***

| **Identity^a^** | **Allele 1** | **Allele 2** | **Pollen donor^c^** | **Stigma recipient^c^** | **SRA** |
| --- | --- | --- | --- | --- | --- |
| d32 | Ah03 | Ah12 | Mor_19_2 | Mor_19_19 | SAMN20844104 |
| d33 | Ah01 | Ah12 | Mor_19_3 | Mor_19_4 | SAMN20844105 |
| d38^d^ | Ah04 | Ah04 | Mor_19_13 | Mor_19_14 | SAMN20844106 |
| d42 | Ah20 | Ah12 | Mor_19_23 | Mor_19_24 | SAMN20844107 |
| d72 | Ah20 | Ah36 | Mor_19_37 | Mor_19_38 | SAMN20844108 |
| d74 | Ah25 | Ah12 | Mor_19_41 | Mor_19_42 | SAMN20844109 |
| d76 | Ah03 | Ah36 | Mor_19_46 | Mor_19_45 | SAMN20844110 |
| d89 | Ah03 | Ah05 | Mor_19_56 | Mor_19_55 | SAMN20844111 |
| d217 | Ah12 | Ah59 | Mor_19_19 | Mor_19_37 | SAMN20844112 |
| d250 | Ah12 | Ah20 | Mor_19_24 | Mor_19_51 | SAMN20844113 |
| d265 | Ah36 | Ah24 | Mor_19_53 | Mor_19_54 | SAMN20844114 |
| d3.1 | Ah24 | Ah25 | Niv_19_4 | Niv_19_5 | SAMN20844115 |
| d10.1^f^ | Ah03 | Ah24 | Niv_19_18 | Niv_19_19 | SAMN20844116 |
| d12.1^f^ | Ah01 | Ah20 | Niv_19_22 | Niv_19_23 | SAMN20844117 |
| d30.1 | Ah02 | Ah01 | Niv_19_58 | Niv_19_59 | SAMN20844118 |
| d48.1 | Ah20 | Ah25 | Niv_19_52 | Niv_19_5 | SAMN20844119 |
| d50.1^e^ | Ah12 | Ah04 | Niv_19_8 | Niv_19_60 | SAMN20844120 |
| d51.1 | Ah12 | Ah04 | Niv_19_54 | Niv_19_7 | SAMN20844121 |
| d191.1^e,f^ | Ah04 | Ah04 | Niv_19_3 | Niv_19_31 | SAMN20844122 |
| d206.1 | Ah22 | Ah25 | Niv_19_42 | Niv_19_5 | SAMN20844123 |
| d205.1^f^ | Ah20 | Ah04 | Niv_19_53 | Niv_19_9 | SAMN20844124 |
| **d208.1^f^** | **Ah03** | **Ah02** | **Niv_19_58** | **Niv_19_18** | **SAMN20844125** |
| **d17.1^f^** | **Ah03** | **Ah01** | **Niv_19_33** | **Niv_19_32^b^** | **SAMN20844126** |
| **d24.1^f^** | **Ah20** | **Ah01** | **Niv_19_47^b^** | **Niv_19_46^b^** | **SAMN20844127** |
| **d29.1^f^** | **Ah12** | **Ah01** | **Niv_19_57^b^** | **Niv_19_56^b^** | **SAMN20844128** |
| **d50.2^f^** | **Ah04** | **Ah04** | **Niv_19_8** | **Niv_19_60** | **SAMN20844129** |
| d266.1 | Ah04 | Ah12 | Niv_19_7 | Niv_19_45 | SAMN20844130 |
| d122 | Ah01 | Ah63 | Pin_16_1 | Pin_8_2 | SAMN20844131 |
| d127 | Ah01 | Ah03 | Pin_5_1 | Pin_4_54 | SAMN20844132 |
| d173 | Ah03 | Ah18 | Pin_5_2 | Pin_16_3 | SAMN20844133 |
| d176 | Ah03 | Ah42 | Pin_15_1 | Pin_9_1 | SAMN20844134 |
| d177 | Ah01 | Ah29 | Pin_16_1 | Pin_5_12 | SAMN20844135 |
| d239 | Ah29 | Ah01 | Pin_4_24 | Pin_8_15 | SAMN20844136 |
| d113 | Ah18 | Ah31 | Tss_3_23 | Tss_22_7 | SAMN20844137 |
| d273 | Ah03 | Ah01 | Tss_23_2 | Tss_22_24 | SAMN20844138 |
| d275 | Ah63 | Ah31 | Tss_23_2 | Tss_14_3 | SAMN20844139 |
| d284 | Ah01 | Ah31 | Tss_21_10 | Tss_5_1 | SAMN20844140 |
| d285 | Ah01 | Ah31 | Tss_21_10 | Tss_3_10 | SAMN20844141 |
| d118 | Ah03 | Ah01 | Ind_9_3 | Ind_8_1 | SAMN20844142 |
| d163 | Ah03 | Ah01 | Ind_8_1 | Ind_1_1 | SAMN20844143 |
| d166 | Ah03 | Ah01 | Ind_6_1 | Ind_15_1 | SAMN20844144 |
| d170 | Ah01 | Ah63 | Ind_9_3 | Ind_15_2 | SAMN20844145 |
| d232 | Ah01 | Ah31 | Ind_18_1 | Ind_10_3 | SAMN20844146 |

***^a^*** *Offspring figured in bold were not used to reconstitute haplotype of parents.* ***^b^*** *Parents were not sequenced.* ***^c^*** *Italics indicate parental haplotypes that were suppressed from the dataset because they had already been phased through another offspring .*  ***^d^*** *The homozygous genotype of d38 was confirmed by genome-wide sequencing.* ***^e^*** *The homozygous genotypes of d191 and d50.1 were confirmed by the proportion of homozygous S-alleles in their offspring found by PCR after cross.* ***^f^*** *The individuals used for crosses in the phenotypic experiments.*

***Supplementary file 1i: Effect of phytopathogen, phytophagous attacks and oxidative stress on the phenotypic traits.***

| **Effect tested** | **Trait** | **Mean with attack (sd)** | **Mean without attack (sd)** | **p value ^a^** |
| --- | --- | --- | --- | --- |
| Phytopathogen | Time first leaf (day) | 15.37 (4.84) | 15.38 (5.24) | 0.49 |
|  | Number of leaves | 13.03 (2.9) | 13.46 (3.71) | 0.23 |
|  | Rosette area (cm²) | 31.93 (27.19) | 37.08 (27.14) | 0.13 |
|  | Mean leave length (cm) | 1.78 (0.78) | 1.91 (0.8) | 0.16 |
|  | St dev leave length (cm) | 0.29 (0.14) | 0.28 (0.13) | 0.38 |
|  | Mean leave width (cm) | 1.42 (0.56) | 1.51 (0.53) | 0.17 |
|  | St dev leave width (cm) | 0.25 (0.11) | 0.27 (0.12) | 0.23 |
|  | Mean leave area (cm²) | 2.94 (2.33) | 3.25 (2.15) | 0.2 |
|  | **Time first flower (day)** | **37.43 (4.56)** | **31.34 (5.55)** | **2e-^03^** |
|  | Number of flowering stems | 12.05 (4.4) | 11.84 (4.62) | 0.05 |
|  | Maximum flowering stem length (cm) | 56.78 (12.04) | 52.55 (11.82) | 0.1 |
|  | Number of flowers by flowering stem | 56.56 (30.99) | 56.22 (34.55) | 0.11 |
|  | **Flowering duration (day)** | **41.44 (8.13)** | **39.51 (7.32)** | **9e-^03^** |
| Phytophagous | Time first leaf (day) | 14.48 (4.4) | 15.56 (5.2) | 0.16 |
|  | Number of leaves | 12.59 (3.76) | 13.44 (3.35) | 0.13 |
|  | Rosette area (cm²) | 32.15 (32.24) | 35.77 (26.16) | 0.27 |
|  | Mean leave length (cm) | 1.68 (0.83) | 1.9 (0.78) | 0.1 |
|  | St dev leave length (cm) | 0.29 (0.1) | 0.28 (0.14) | 0.36 |
|  | **Mean leave width (cm)** | **1.29 (0.55)** | **1.51 (0.53)** | **0.03** |
|  | **St dev leave width (cm)** | **0.22 (0.1)** | **0.27 (0.12)** | **0.03** |
|  | Mean leave area (cm²) | 2.59 (2.3) | 3.25 (2.19) | 0.08 |
|  | **Time first flower (day)** | **34.78 (6.93)** | **33.36 (4.92)** | **0.04** |
|  | Number of flowering stems | 10.78 (4.76) | 12.15 (4.55) | 0.34 |
|  | **Maximum flowering stem length (cm)** | **53.53 (9.81)** | **54.24 (12.2)** | **0.05** |
|  | Number of flowers by flowering stem | 47.46 (25.45) | 58.12 (34.46) | 0.17 |
|  | Flowering duration (day) | 37.56 (8.1) | 40.76 (7.76) | 0.45 |
| Oxidative stress | Time first leaf (day) | 14.92 (3.93) | 15.46 (5.28) | 0.34 |
|  | **Number of leaves** | **10.04 (3.88)** | **13.93 (2.96)** | **1e-^04^** |
|  | **Rosette area (cm²)** | **11.79 (16.54)** | **39.64 (26.56)** | **1e-^04^** |
|  | **Mean leave length (cm)** | **1.05 (0.49)** | **2.02 (0.74)** | **1e-^04^** |
|  | St dev leave length (cm) | 0.25 (0.1) | 0.29 (0.14) | 0.09 |
|  | **Mean leave width (cm)** | **0.88 (0.31)** | **1.59 (0.5)** | **1e-^04^** |
|  | **St dev leave width (cm)** | **0.23 (0.12)** | **0.27 (0.12)** | **0.04** |
|  | **Mean leave area (cm²)** | **1.05 (0.98)** | **3.54 (2.17)** | **1e-^04^** |
|  | Time first flower (day) | 31.77 (5.6) | 33.95 (5.25) | 0.06 |
|  | Number of flowering stems | 10.38 (4.85) | 12.21 (4.54) | 0.44 |
|  | Maximum flowering stem length (cm) | 50.3 (13.1) | 54.85 (11.71) | 0.11 |
|  | Number of flowers by flowering stem | 53.97 (55.98) | 56.8 (28.05) | 0.21 |
|  | Flowering duration (day) | 37.35 (6.5) | 40.78 (7.94) | 0.08 |

*^a^ The P value represents the proportions of the distribution with the expected difference of mean between individuals with and without attack after 10,000 random resamples equal to or less than the value observed. Significant values are represented in bold.*

***Supplementary file 1j: Difference on the phenotypic trait variation between the two families for each allele tested.***

| ***Allele*** | ***Family (Male/Female)*** | ***Trait (unit)*** | ***Mean (sd)*** | ***p value^a^*** |
| --- | --- | --- | --- | --- |
| *Ah01* | *1 (d29.1/d24.1)* | ***Time first leaf (day)*** | ***20.8 (5.43)*** | ***2e^-03^*** |
|  |  | *Number of leaves* | *11.9 (3.75)* | *0.09* |
|  |  | *Rosette area (cm²)* | *24.9 (25.58)* | *0.11* |
|  |  | *Mean leaf length (cm)* | *1.53 (0.79)* | *0.08* |
|  |  | *St dev leaf length (cm)* | *0.21 (0.09)* | *0.03* |
|  |  | *Mean leaf width (cm)* | *1.4 (0.58)* | *0.33* |
|  |  | *St dev leaf width (cm)* | *0.24 (0.14)* | *0.32* |
|  |  | *Mean leaf area (cm²)* | *2.47 (1.93)* | *0.17* |
|  |  | *Time first flower (day)* | *28.8 (5.29)* | *0.44* |
|  |  | ***Number of flowering stems*** | ***8 (4.07)*** | ***0.02*** |
|  |  | ***Maximum flowering stem length (cm)*** | ***40.65 (6.76)*** | ***0.02*** |
|  |  | ***Number of flowers by flowering stem*** | ***35.85 (10.72)*** | ***0.04*** |
|  |  | ***Flowering duration (day)*** | ***40.35 (12.45)*** | ***8e^-03^*** |
| *Ah01* | *2 (d12.1/d29.1)* | ***Time first leaf (day)*** | ***18.76 (5.57)*** | ***1e^-04^*** |
|  |  | *Number of leafs* | *13.45 (3.22)* | *0.42* |
|  |  | *Rosette area (cm²)* | *31.99 (22.61)* | *0.25* |
|  |  | *Mean leaf length (cm)* | *1.74 (0.71)* | *0.19* |
|  |  | *St dev leaf length (cm)* | *0.29 (0.1)* | *0.42* |
|  |  | *Mean leaf width (cm)* | *1.44 (0.51)* | *0.34* |
|  |  | *St dev leaf width (cm)* | *0.29 (0.11)* | *0.11* |
|  |  | *Mean leaf area (cm²)* | *2.84 (1.89)* | *0.21* |
|  |  | ***Time first flower (day)*** | ***29.86 (5.43)*** | ***0.04*** |
|  |  | *Number of flowering stems* | *9.59 (3.54)* | *0.32* |
|  |  | ***Maximum flowering stem length (cm)*** | ***34.85 (9.35)*** | ***1e^-04^*** |
|  |  | ***Number of flowers by flowering stem*** | ***30.27 (25.45)*** | ***1e^-04^*** |
|  |  | *Flowering duration (day)* | *31.62 (7.17)* | *0.06* |
| *Ah03* | *3 (d208.1/d17.1)* | *Time first leaf (day)* | *13.33 (5.27)* | *0.22* |
|  |  | *Number of leaves* | *12.05 (4.25)* | *0.17* |
|  |  | *Rosette area (cm²)* | *31.23 (25.7)* | *0.35* |
|  |  | *Mean leaf length (cm)* | *1.67 (0.78)* | *0.32* |
|  |  | *St dev leaf length (cm)* | *0.28 (0.15)* | *0.42* |
|  |  | *Mean leaf width (cm)* | *1.39 (0.62)* | *0.5* |
|  |  | *St dev leaf width (cm)* | *0.24 (0.13)* | *0.42* |
|  |  | *Mean leaf area (cm²)* | *2.87 (2.24)* | *0.45* |
|  |  | ***Time first flower (day)*** | ***34.81 (7.87)*** | ***0.02*** |
|  |  | ***Number of flowering stems*** | ***7.67 (3)*** | ***3e^-04^*** |
|  |  | ***Maximum flowering stem length (cm)*** | ***44.4 (13.87)*** | ***0.03*** |
|  |  | *Number of flowers by flowering stem* | *61.55 (46.06)* | *0.06* |
|  |  | ***Flowering duration (day)*** | ***39 (6.6)*** | ***5e^-03^*** |
| *Ah03* | *4 (d10.1/d17.1)* | Time first leaf (day) | 18 (6.2) | 0.12 |
|  |  | Number of leaves | 12.33 (4.46) | 0.22 |
|  |  | **Rosette area (cm²)** | **17.71 (11.23)** | **0.04** |
|  |  | Mean leaf length (cm) | 1.52 (0.53) | 0.14 |
|  |  | St dev leaf length (cm) | 0.23 (0.07) | 0.14 |
|  |  | Mean leaf width (cm) | 1.23 (0.35) | 0.13 |
|  |  | St dev leaf width (cm) | 0.23 (0.08) | 0.23 |
|  |  | Mean leaf area (cm²) | 2.01 (1.34) | 0.1 |
|  |  | Time first flower (day) | 33.33 (4.23) | 0.09 |
|  |  | Number of flowering stems | 11.67 (4.23) | 0.22 |
|  |  | Maximum flowering stem length (cm) | 57.25 (3.69) | 0.34 |
|  |  | Number of flowers by flowering stem | 75.05 (29.69) | 0.12 |
|  |  | Flowering duration (day) | 45.5 (8.67) | 0.33 |
| *Ah04* | *5 (d205.1/d191.1)* | ***Time first leaf (day)*** | ***13.42 (3.28)*** | ***1e^-04^*** |
|  |  | *Number of leaves* | *13.45 (2.97)* | *0.32* |
|  |  | *Rosette area (cm²)* | *36.29 (27.39)* | *0.31* |
|  |  | *Mean leaf length (cm)* | *1.92 (0.8)* | *0.22* |
|  |  | *St dev leaf length (cm)* | *0.29 (0.15)* | *0.34* |
|  |  | *Mean leaf width (cm)* | *1.54 (0.56)* | *0.11* |
|  |  | *St dev leaf width (cm)* | *0.27 (0.13)* | *0.15* |
|  |  | *Mean leaf area (cm²)* | *3.36 (2.41)* | *0.14* |
|  |  | *Time first flower (day)* | *35.1 (4.66)* | *0.33* |
|  |  | *Number of flowering stems* | *12.87 (4.16)* | *0.32* |
|  |  | ***Maximum flowering stem length (cm)*** | ***62.96 (9.48)*** | ***1e^-04^*** |
|  |  | ***Number of flowers by flowering stem*** | ***70.13 (32.5)*** | ***1e^-04^*** |
|  |  | *Flowering duration (day)* | *42.44 (7.05)* | *0.3* |
| *Ah04* | *6 (d205.1/d50.2)* | *Time first leaf (day)* | *14.31 (4.36)* | *0.30* |
|  |  | *Number of leaves* | *13.5 (3.9)* | *0.13* |
|  |  | ***Rosette area (cm²)*** | ***44.1 (33.48)*** | ***0.02*** |
|  |  | ***Mean leaf length (cm)*** | ***2.04 (0.89)*** | ***0.04*** |
|  |  | *St dev leaf length (cm)* | *0.29 (0.12)* | *0.21* |
|  |  | *Mean leaf width (cm)* | *1.38 (0.51)* | *0.36* |
|  |  | ***St dev leaf width (cm)*** | ***0.21 (0.09)*** | ***0.03*** |
|  |  | *Mean leaf area (cm²)* | *3.33 (2.27)* | *0.21* |
|  |  | ***Time first flower (day)*** | ***31.92 (3.66)*** | ***2e^-03^*** |
|  |  | ***Number of flowering stems*** | ***15.77 (5.25)*** | ***1e^-04^*** |
|  |  | *Maximum flowering stem length (cm)* | *60.01 (9.34)* | *0.07* |
|  |  | ***Number of flowers by flowering stem*** | ***43.51 (15.45)*** | ***5e^-04^*** |
|  |  | ***Flowering duration (day)*** | ***39.96 (7.38)*** | ***0.04*** |

*^a^ The P value represents the proportions of the distribution with the expected difference of mean between each family and the other individuals after 10,000 random resamples equal to or less than the value observed. Significant values are represented in bold.*
